# Getting more than “claps”: incentive preferences of voluntary community-based mobilizers in Tanzania

**DOI:** 10.1186/s12960-019-0438-5

**Published:** 2019-12-17

**Authors:** Maryse Kok, Dinu Abdella, Rose Mwangi, Mengi Ntinginya, Ente Rood, Jennifer Gassner, Kathryn Church, Nkemdiri Wheatley

**Affiliations:** 1 KIT Royal Tropical Institute, P.O. Box 95001, 1090 HA Amsterdam, The Netherlands; 2 Institute of Public Health, Kilimanjaro Christian Medical University College (KCMC), P.O. Box 2240, Moshi, Tanzania; 3 Marie Stopes Tanzania, P.O. Box 7072, Das es Salaam, Tanzania; 4 Marie Stopes International, 1 Conway Street Fitzroy Square, London, W1T 6LP United Kingdom

**Keywords:** Community health worker, Motivation, Performance, Tanzania

## Abstract

**Background:**

Marie Stopes Tanzania works with a voluntary cadre of 66 community-based mobilizers (CBMs), who are tasked with raising awareness, generating demand and providing referral to potential clients for family planning, comprehensive post-abortion care and cervical cancer screening. CBMs extend the reach of urban clinics to peri-urban communities, enhancing access to sexual and reproductive health services. In an effort to optimize performance of CBMs, a study was conducted to explore the drivers of CBM motivation and inform the design of an incentive scheme.

**Methods:**

Three focus group discussions with 17 CBMs and 11 interviews with CBM supervisors and managers were conducted in three clinics and the head office. After thematic analysis of transcripts, findings on motivational factors were discussed in a reflection workshop and informed the development of a discrete choice experiment (DCE) involving 61 CBMs as respondents. The DCE included eight choice questions on two incentive schemes, each consisting of five attributes related to remuneration, training, supervision, benefits and identification. For each attribute, different incentive options were presented, based on the outcomes of the qualitative assessment. The DCE results were analysed using conditional logistic regression.

**Results:**

A variety of factors motivated CBMs. Most CBMs were motivated to conduct their work because of an intrinsic desire to serve their community. The most mentioned extrinsic motivational factors were recognition from the community and supervisors, monthly allowance, availability of supporting materials and identification, trainings, supervision and feedback on performance. Recommendations for improvement were translated into the DCE. Incentive attributes that were found to be significant in DCE analysis (*p* < 0.05), in preference order, were carrying an ID card, bi-monthly training, supervision conducted via both monthly meetings at clinics and visits from the head office, and a monthly flat rate remuneration (over pay for performance).

**Conclusion:**

Despite the recognition that being a CBM is voluntary, incentives, especially those of non-financial nature, are important motivators. Incentive schemes should include basic compensation with a mix of other incentives to facilitate CBMs’ work and enhance their motivation. Programme designs need to take into account the voices of community-based workers, to optimize their performance and service delivery to communities they serve.

## Background

Community health workers (CHWs) form an instrumental part of health systems in low- and middle-income countries. They mostly focus on health promotion and disease prevention and form the connection between communities and the health sector, partly through referral. There are many different types and names of CHWs, and their position in health systems varies, from fully integrated and salaried workers with multiple tasks to voluntary workers, who are often attached to specific health programmes [1, 2].

While many governments are in the process of establishing a formalized cadre of CHWs, in many countries, a multitude of different types of CHWs are present, who are often deployed by non-governmental organizations (NGOs). This is also the case in Tanzania, where the government is in the process of implementing the National Community Based Health Care Strategic Plan 2014–2020, which includes revival and integration of paid CHWs, trained for 1 year, into the health workforce [3]. However, this process is on hold, and at the moment, voluntary CHWs of different types still dominate the community health landscape.

One of the voluntary CHW cadres in Tanzania are community-based mobilizers (CBMs), deployed by Marie Stopes Tanzania (MST). This cadre, different from many others, works in urban and peri-urban areas where they are tasked with raising awareness, generating demand and providing referral to potential clients for MST services, such as family planning, comprehensive post-abortion care and cervical cancer screening. CBM selection criteria include being 18 years or above, completion of primary school or a higher education level, and being known by and having understanding of their community. Sixty-six CBMs serve catchment areas of 11 clinics, which cover the four regions of Tanzania (a group of six CBMs per clinic). Like in other countries of operation, CBMs serve at a voluntary basis, though they receive a small stipend (40 000 Tsh/17.50 USD per month). Their average time investment per week varies: the majority reports spending 1 to 4 days per month on their work, the rest reports spending 5 days or more.

In recent years, governments, programmers and researchers have shown interest in optimizing motivation and performance of CHWs. The design of CHW programmes, whether integrated in the health system or related to specific NGO programmes, can influence CHWs’ motivation, which is an instrumental component of performance [4]. Evidence has shown that CHW performance can be sub-optimal, because of inadequate training and supervision, insufficient incentives and lack of supplies and logistical support [2]. An earlier study on incentives and disincentives for CHWs pointed towards the problem of high attrition rates of voluntary CHW programmes, leading to high costs and lack of continuity in relationships between CHWs and communities. The study concluded that a complex set of factors influence CHW motivation and attrition and that these factors differ per context [5].

Motivation can be defined as “an individual’s degree of willingness to exert and maintain an effort towards organizational goals” [6]. It can be seen as a transactional process between an individual and the work environment, influenced by the broader context. Factors that influence motivation can be categorized as extrinsic and intrinsic [6]. Extrinsic motivational factors can be further categorized into financial, non-financial and in-kind [7]. While financial incentives, such as stipends, allowances and performance-based financial rewards are found to be important for motivation of voluntary and paid CHWs, non-financial incentives are often found to be as important [8–10]. A study on voluntary CHWs in Tanzania found that CHWs were driven by intrinsic motivation: the love for the work, the commitment towards the public service and the community, and the desire to help themselves and their families. Furthermore, non-financial incentives, in the form of moral family support, respect and recognition from the community and support from the organization or programme (such as training, supervision and material support) influenced CHW motivation [10].

Some scholars have debated that voluntary CHWs have higher intrinsic motivation and commitment towards their communities than officially paid CHWs [11, 12]. Payment of salaries could lead to demotivation of CHWs when expectations are not met, cause tension when different cadres of CHWs compare their salaries [5] and weaken internalized extrinsic motivation or even intrinsic motivation [13].

To date, many CHW programmes, including the CBM programmes of Marie Stopes International, still face challenges with poor motivation and retention. Existing evidence on which incentive packages improve CHWs’ motivation and performance in various contexts is still limited [14]. Therefore, a study was conducted to explore the drivers of CBM motivation in Tanzania and inform the design of an (adjusted) incentive scheme that could result in improved CBM performance. The study aimed to contribute to strengthening MST’s CBM operations, but could also provide insights for the revived CHW programme in Tanzania and other countries.

## Methods

We conducted a mixed-methods study in November–December 2017. The first phase included focus group discussions (FGDs) with CBMs, key informant interviews (KIIs) with CBM supervisors and managers and a reflection workshop with MST staff. This fed into a second phase, comprising a discrete choice experiment (DCE).

The first phase was conducted in three MST clinics: Mabibo (Dar es Salaam region), Kahama (Shinyanga region) and Mbeya (Mbeya region), of which the first presents an urban setting and the latter two present peri-urban settings. Besides the geographical location and setting, the selection of the three clinics was based on a combination of how the CBMs were performing in relation to how the clinic was performing, based on the number of clients (referred). This combination involved: a clinic that was considered underperforming and its CBMs were underperforming; a clinic that was considered well-performing and its CBMs were well-performing; and a clinic that was considered underperforming and its CBMs were well-performing.

The FGDs, in which 17 CBMs participated, explored how CBMs experienced their tasks, which factors motivated them in their work, what they valued in the current incentive scheme and what they would prefer as possible new incentives. We used a broad definition of incentives: positive or negative, intrinsic or extrinsic factors influencing CHW motivation and volunteerism [5]. Additionally, KIIs were conducted with staff who were directly involved with the CBMs. This included four staff from the head office, two clinic managers and three CBM supervisors who also work at the clinic level. Two KIIs were carried out with representatives of the District Health Office who were knowledgeable about CBMs and other CHWs in the district. Topic guides were pre-tested in a clinic not included in the study, after which small adjustments were made, which mainly concentrated on the expression of terms such as “motivation” and “incentives” in Swahili. Data were collected by a team of two researchers experienced in qualitative research methods.

FGDs and KIIs were digitally recorded, transcribed and, when applicable, translated. A combination of a deductive approach, using pre-existing themes based on literature [6, 7] and topic guides, and an inductive approach, which allowed new themes to emerge from the data [15], was used to develop a coding framework. Transcripts were coded and data further analysed, “charted” in themes and subthemes and summarized in narratives for each theme and subtheme.

Preliminary results gave rise to questions about motivation and performance of CBMs that were discussed in a reflection workshop with MST and Marie Stopes International staff. During this workshop, existing and preferred new incentives that came out of the qualitative component were also discussed. Based on expected effect on motivation and budget availability, various feasible programmatic incentive scheme options were identified, which were quantified using a discrete choice experiment (DCE) conducted during the second phase of the project.

A DCE comprises a quantitative analytic technique used for eliciting stated preferences from a predefined set of alternatives. Using an experimental design, respondents are presented with pairs of hypothetical scenarios described in terms of combinations of attributes that can take different levels [16]. In this study, we used a DCE to elicit the incentive preferences of CBMs. The attributes were based on the major factors influencing extrinsic motivation of CBMs as emerged from phase 1. We included five attributes, each having two levels (Table 2) which were used to develop a questionnaire with eight assignments in which CBMs were to choose between two sets of possible incentives (Additional file 1). The five attributes with two levels each resulted in a total of 32 choice profiles and 512 different choice scenarios. A factorial orthogonal design was used to test for the main effects of different attributes on CBM preferences [17], resulting in a simple choice set of eight questions. The minimum sample size for the DCE to test for the main attribute effects was 50 [18, 19]. The goodness of the fit of the model was assessed by means of the likelihood ratio of the model including the choice attributes over the unrestricted model using and intercept only.

The DCE was pre-tested to assess the selection of attributes and their levels, respondents’ understanding of the questionnaire’s content and process and feasibility of self-administration in this context [20, 21]. The pre-test did not lead to changes in the questionnaire, but led to slight adjustments in the instructions given to the CBMs prior to filling in the questionnaire. The final choice set of eight questions were administered to 61 CBMs, this included the CBMs who participated in the pre-test. Five of the 66 CBMs working for MST were not available during data collection. Questionnaires were administered to CBMs sitting together in a room, but with each CBM completing the questionnaire individually on paper (i.e. self-administration), after having received instructions from the researchers (in four clinics) or MST head office staff (in seven clinics).

DCE data from the completed questionnaires were first double entered into Excel and inconsistencies between sequential edits were corrected before data analysis. Data analysis was conducted using R 3.3.2. language for statistical analysis [22]. Respondents selected their preferred scenario between the two sets of possible incentives, and response data were analysed with conditional logistic regression models to estimate the influence of each attribute on their choice, where *p* < 0.05 was considered as significant.

## Results

CBMs, their managers and supervisors reported a variety of motivational factors (Table 1: whether these factors were present, as perceived by most CBMs, is indicated by + (present), +/− (partly present and partly not present) and − (not present) respectively). The qualitative findings did not reveal any differences in perceptions between participants of the three selected clinics.

**Table 1:** Motivational factors as reported by CBMs and other study participants

Category of motivational factors	Main reported motivation factors
Intrinsic motivational factors	Passion to share information (+) Wish to help people (+)
Extrinsic motivational factors	
Financial	*Posho* [allowance] (+/−)
Non-financial	Recognition from clinic management (+) Recognition from head office (−) Recognition and respect from community (+) Supervision (+) Performance appraisal (−) Trainings, seminars, meetings (+/−) Personal development, gained knowledge (+)
In-kind	Tea, soda (+) Uniform in the form of blue T-shirts (+/−) Working materials - brochures, condoms (+/−)

### Intrinsic motivational factors

Intrinsic motivational factors were linked to reasons for becoming a CBM. When asked why they agreed to become CBM, statements like “passion about sharing information with the community on important issues like cervical cancer screening and family planning” and “wanting to share with others about the friendly quality services in a clean environment” were common among CBMs.

### Financial incentives

CBMs were given a monthly allowance of 40 000 TSh (17.50 USD). It was transferred to CBMs on the condition that country-wide, all CBM monthly reports were submitted to the head office. All CBMs reported that they did not know whether there was a financial reimbursement or incentive before becoming CBMs: they only came to learn about the allowance after their recruitment. Similarly, many stated that they did not know what voluntarism entailed until they started: “We didn’t know but when we started, that is when we were told it is voluntary… I just agreed, but I didn’t know what volunteering is.”

The majority stated that what they get is just *posho* [allowance]—as opposed to a monthly salary. The topic of posho was raised in all FGDs, some saw it as reimbursement for personal costs, others for transport, or both. All CBMs mentioned that receiving posho motivated them, and they wished the posho to be raised: “I feel good about myself with what I have achieved with my work for MST, until I arrive home at the end of the day and realise I don’t even have sugar for my children. Then I wish, I could get a little bit of a compensation at least when I am doing well.” It was also emphasized that the posho should come in time: the condition of all CBM monthly reports having to be in before payment caused delays.

A few FGD participants reported that CBMs were believed to earn a lot of money and that the community did not believe that the CBM work is voluntary. This could potentially hinder CBM motivation and performance, because of mistrust from the side of the community.

### Non-financial incentives

In terms of non-financial motivational factors, motivation was first reported to come from within the organization. For instance, CBMs looked at appreciation from the clinic staff as a motivational factor. One CBM said: “We get Makofi [claps] when we do well” and another said “We feel welcomed at the clinic, sometimes even given chai and chapatti and we walk around freely”*.* Feeling accepted as part of the clinic and being surrounded by friendly staff, who are smiling and provide a good service for clients, were seen as strong motivating factors. However, the same recognition was not felt from the side of the head office, who were reported to seldom directly interact with CBMs. The head office staff was not aware that their attention to and interactions with CBMs were a motivational factor for CBMs.

Generally, the communities seemed to trust the CBMs and treated them with respect, which was positively influencing motivation: “They [the community] have positive reactions, because the kind of the service we offer is good enough for them to want to come back. And besides coming back [to the clinic], they also advertise about us.” However, in one clinic, the CBMs reported that some community members ridiculed them by claiming they were prostitutes, which seemed related to their task of promoting contraception, including condoms. CBMs also reported that there were misconceptions about the health services promoted, for example contraceptive pills were sometimes believed to affect the female’s sexual libido and cervical cancer screening was sometimes seen as an interference with a woman’s privacy. The misconceptions of communities were not necessarily reported to be demotivating, rather making the work as a CBM challenging and worthwhile.

Most CBMs could not exactly define what they understood by supervision beyond the monthly meetings that they had with their supervisor. In most cases, they looked at supervision as a welcome idea that could motivate them: “There is no problem with being supervised… it is better because if I am asked a technical question, it will be easier to get an answer from the supervisor.” Supervision was also believed to lead to community trust: “Because it helps to show that we belong to MST and the community trusts us in addition to responding to questions we are not able to answer.” Performance appraisal was not brought up by CBMs, while supervisors mentioned that when they occasionally accompanied CBMs in monthly awareness-raising activities (*vikundi* visits), they filled a checklist and provided feedback on performance.

CBMs reported to value their initial training, but the orientation at the clinic that followed was found to be too short, and they said they needed more refresher trainings. “Maybe, the training can be done once after every three months. We can sit in a room where we are taught by the nurse about family planning, so that when a client asks we are able to answer.” There was a general appreciation of the (one-time) regional training that brought CBMs from different regions together. Technical knowledge shared at the regional training offered social prestige, self-satisfaction and gained the CBMs respect in the community. Besides technical knowledge from trainings, some CBMs also referred to personal knowledge gained by being a CBM. “I meet other people”, “I build my confidence”, “I can speak publicly”, and “I gain additional knowledge from the trainings” were common statements portraying non-financial motivating factors. CBMs did not refer to trainings being financial incentives as well, although for some trainings they did receive transport allowance and per diems. Career perspectives were not brought up by CBMs as well as their supervisors.

### In-kind incentives

CBMs reported that they occasionally received tea and soda in the clinic, which served as a motivational factor. It was common across all FGDs for CBMs to ask for (blue) T-shirts for better identity and bags. “What I will request is that a uniform needs to be considered… The white T-shirt is only one but you cannot wear the white [or the same] T-shirt the whole week.” Some CBMs mentioned the importance of identity cards. CBMs furthermore stressed the importance of having brochures and condoms for distribution, which was echoed by a supervisor: “Maybe working tools like bags with MST logo, or bracelet because there are times they go for community mobilization and you find that people are not listening; but when you have some promotion materials the outcome can be much better.”

### Towards an adjusted incentive scheme—CBMs’ incentive preferences

Following the contributions of CBMs, their supervisors and MST staff, the most suggested areas related to improving CBM motivation were related to remuneration, training, supervision, benefits and identification. During the refection workshop, these five attributes and the formulation of two (feasible) levels per attribute were discussed, and Table 2 shows the final DCE attributes and levels used in the DCE questionnaire (Additional file 1). It should be noted that timeliness of payment and appropriate pre-service training were agreed upon as mandatory management structures that need to be in place and therefore they were not presented in the DCE.

**Table 2:** Attributes and levels used in the DCE

Attribute	Remuneration	Training	Supervision	Benefits	Identification
Level 1	60 000 TSh/month	CBMs to attend a seminar at the clinic on a specific health topic every 2 months.	Monthly progress review meetings including receiving monthly progress report + clinic manager or clinic administrative assistant accompany all monthly awareness-raising activities and give immediate feedback to CBMs.	Once every 6 months, the best performing CBM at your clinic gets the opportunity to visit another clinic.	CBMs will receive two blue Marie Stopes T-shirts + Marie Stopes bag once.
Level 2	50 000 TSh/month + 10 000 TSh extra for every 5 clients referred by the CBM group.	CBMs to attend a seminar at the clinic on a specific health topic every 6 months.	Monthly progress review meetings + MST head-office visits CBMs in the field every 6 months and a group picture is taken.	Once every 6 months, the best performing group of CBMs in Tanzania gets a health insurance covering Marie Stopes services of 50 000 TSh for each person.	CBMs will receive two blue Marie Stopes T-shirts + Marie Stopes bag + ID card once.

A total of 61 CBMs filled the DCE questionnaire, including the CBMs who participated in the pre-test. All CBMs had at least primary education and the majority had secondary education level. Of the 61 CBMs who filled the questionnaire, 72.1% were female and 27.9% were male. The average age was 29.5 years. Of the 73.8% (*n* = 45) CBMs who provided their years of service, the average was 2 years and 2 months, with the shortest years of services being 1 month and the longest 9 years.

The results of the data analysis (Table 3) show that the means of identification and the training package which is offered to CBMs affected the choice of preference most strongly. The univariate models show that an incentive scheme including two T-shirts, a bag and an identity card was preferred over a package with two T-shirts and a bag alone (OR [95%CI] = 1.64[1.28–2.09]). In addition, respondents showed a clear preference for schemes which include capacity building seminars at least once every 2 months (OR [95%CI] = 0.42[0.33–0.54]). With regard to remuneration, CBMs preferred the “flat” amount of 60 000 TSh (26 USD) per month above the flat amount of 50 000 TSh (21.50 USD) plus a “pay for performance” component (OR [95%CI] = 0.62[0.49–0.79]). As for supervision, monthly progress review meetings at the clinic level combined with the head office visiting CBMs in the field every 6 months was preferred over more intensified supervision solely at the clinic level (OR [95%CI] = 1.34[1.05–1.71]). Finally, respondents indicated to have a small (not significant) preference for those schemes which include that once every 6 months, the best performing CBM at each clinic gets the opportunity to visit another clinic, as compared to the best performing group of CBMs getting the benefit of a health insurance (OR [95%CI] = 0.91[0.72–1.16]). These results are consistent when a multivariate model including all attributes was fitted to the data (Likelihood ratio test = 87.65, *p* < 0.0001). As the questionnaire was designed to optimize the efficiency of estimation of the main attribute effects, no apparent interactions were found. In addition, no differences in outcomes were observed by respondents’ characteristics.

**Table 3:** Conditional logistic regression results: CBMs’ incentive preferences in Tanzania, December 2017

Attribute	Level	Univariate model				Multivariate model			
		OR	LCI	UCI	p	OR	LCI	UCI	p
Remuneration	60 K	1	–	–	–	–	–	–	–
	50 K + 10 K bonus	0.62	0.49	0.79	0.000 13	0.6	0.46	0.77	0.000 076
Training	Seminar 2 months	1	–	–	–	–	–	–	–
	Seminar 6 months	0.42	0.33	0.54	5.6E−12	0.4	0.31	0.52	2.5E−12
Supervision	Monthly + immediate feedback	1	–	–	–	–	–	–	–
	Monthly + 6 months MST visit	1.34	1.05	1.71	0.019	1.37	1.06	1.77	0.016
Benefits	Best CBM visit other clinic	1	–	–	–	–	–	–	–
	Best CBM health insurance	0.91	0.72	1.16	0.46	0.92	0.71	1.19	0.54
Identification	Two shirts + bag	1	–	–	–	–	–	–	–
	Two shirts + bag + ID card	1.64	1.28	2.09	0.000 076	1.71	1.32	2.21	0.000 039

*OR* odds ratio, *LCI/UCI* lower and upper 95% confidence interval respectively, *p* = value of model coefficient, bold results are significant (*p* < 0.05)

## Discussion

This study provides evidence on preferred incentive options of CBMs working for MST. This evidence will be used to pilot and evaluate different incentive schemes at a later stage, with a focus on motivation and performance indicators. While providing direction to future incentive schemes of MST, the findings might be useful for programmes including voluntary CHWs with similar tasks in other settings.

As found in other studies [23, 24], the voluntary CBMs in this study preferred a combination of financial and non-financial incentives, where non-financial incentives, particularly identification—a “hardware element” that was found to strongly influence “software elements” such as community trust and recognition [25], training and supervision were preferred above financial incentives. The recently published World Health Organization (WHO) guideline on health policy and system support to optimize CHW programmes also recommends a mix of different types of incentives [14, 26]. Despite a low certainty of evidence, the WHO strongly recommends remuneration of practising CHWs for their work “with a financial package commensurate with the job demands, complexity, number of hours, training and roles that they undertake” (14, p., 47). This recommendation was partly made in the light of the international agenda on decent work, sustainable development goal 8, and equity considerations, as CHWs are often female. At the same time, the WHO recognizes that volunteers who spend limited time on the job and have other sources of income will continue to play a role in health systems [14]. Volunteers are often driven by intrinsic motivational factors, such as religious or moral duty and altruistic concerns for others and extrinsic, non-financial incentives such as community respect and recognition of professional health workers [27–29]. However, there can be ambivalence in motivation: when (changed) circumstances make volunteers uncertain in achieving food security, or when an improved socio-economic status is experienced for themselves and their families, volunteers may start to request remuneration [30]. This shows the necessity for policy makers and programme managers to assess incentive preferences over time. In addition, this study shows that clarity about the voluntary nature of the CHW work, including about the types of incentives offered, is important to maintain community trust and respect.

The importance of non-financial incentives, such as (refresher) training and supportive supervision, is also stressed by the WHO guideline [14, 31]. While—according to the definition of incentives used in this paper—these programme elements influence CHW motivation and volunteerism, they can be seen as “job enablers” instead of incentives, as essentially they constitute the basic enabling environment that CHW programmes should provide for CHWs to be able to execute their tasks [32]. Identification in the form of an ID card can motivate CHWs to perform better because it increases recognition from the community [25], but in the case of CBMs in Tanzania, it could be argued that it also provides safety in areas where misconceptions about the role or messages of CBMs are prevalent in some areas. The non-financial incentive of career perspective was not brought up during FGDs with CBMs, contrary to findings from other studies [4]. This could indicate that CBMs, their supervisors and managers regarded the programme as truly voluntary, where the limited time investment of CBMs did not trigger the wish for future career advancement.

This study found that CBMs did not prefer a pay-for-performance option over a flat rate payment scheme. This could be because systems of performance appraisal were found to be weak, and performance was currently measured only in the number of referrals made, possibly ignoring other important elements of the work of a CBM [23, 33]. It could also be that despite a recommendation for a pay-for-performance option was made by a few CBMs in the first phase of this study, some CBMs did not understand the pay-for-performance option, because they did not have experience with the concept. Although a few studies have shown that performance-based incentives for CHWs could improve case finding for tuberculosis [34, 35], other studies found that non-incentivized tasks got neglected [23] and one study argues that performance-based incentives do not provide voluntary CHWs enough financial security and impede CHWs’ rights [36]. Therefore, the preference of the “flat amount” could also be related to the value of having financial security. The new WHO guideline makes a conditional suggestion not to pay CHWs exclusively or predominantly according to performance-based incentives (14, p., 47). It should be noted that the benefits attribute in the DCE in this study consisted of rewarded benefits for best performing individual CBMs or groups of CBMs. As such, they could be seen as non-financial or in-kind incentives based on performance. However, the study did not find a significant preference for these kinds of incentives.

DCEs have been used in health systems research, for example to elicit employment preferences of professional health workers [37, 38]. One study already found that DCEs can also be used for voluntary CHWs [16]. While a key limitation of DCEs is that presented attributes are hypothetical, the DCE was designed to include feasible, future options for CBMs’ incentives. Our DCE was conducted among a specific and relatively homogenous group of CBMs. Findings are not generalizable, but could provide insights about incentive preferences of voluntary CHWs with similar tasks in other settings. The study does not provide insight into the preferred amount of (flat) payment. As the sample size was small, no differences in outcomes were observed by for example sex or clinic location. To increase the validity of our research findings, the included attributes and their levels were based on a small-scale qualitative study (phase 1) to gain insight into CBMs’, their supervisors’ and manager/decision makers’ experiences and opinions [20, 21]. We decided to include (only) five attributes, to ensure that CBMs were able to consider all attributes listed while making their choice [39]. For each of the attributes, we used a combination of increments and mutually exclusive options (levels 1 and 2). There could have been misinterpretations among the CBMs about attributes and their levels, but the pre-test showed that the CBMs were able to fill the DCE and made reasoned and deliberate choices, even in the absence of probing. We also limited the number of assignments to eight to avoid CBMs becoming overwhelmed. This may have affected our ability to detect true underlying preferences [16]. During data collection, we did not use multiple versions of the questionnaire, and therefore, positional bias in responding to the choice sets could have occurred. In seven of the 11 clinics, the DCE was introduced by MST head office staff. Although this staff did not have direct relations with CBMs, it could be that CBMs were influenced by their presence while filling the questionnaire. Despite this, the outcomes of the DCE, showing the importance of non-financial incentives, do correspond with the qualitative component of the study.

## Conclusion

Despite the recognition that being a CBM is voluntary, incentives, especially those of non-financial nature, are important motivators and can potentially improve CBM performance. Incentive schemes for voluntary CBMs should include basic financial compensation with a mix of other incentives, including identification, regular refresher trainings and supportive supervision from the clinic and head office level.

Voices of CHWs should be heard to optimize CHW programme designs, improve CHW motivation and performance and prevent CHW attrition. This study shows that voluntary CHWs are well able to indicate their incentive preferences via a DCE. Continuous attention is needed to ensure incentives are in line with CHWs’ roles and tasks and broader socio-economic context.

## Supplementary information

**Additional file 1.** DCE questionnaire.

## Data Availability

The data that support the findings of this study are available from Marie Stopes International, but restrictions apply to the availability of these data, which were used under license for the current study, and so are not publicly available. Data are however available from the authors upon reasonable request and with permission of Marie Stopes International.

## Abbreviations

CBM: Community-based mobilizer
CHW: Community health worker
DCE: Discrete choice experiment
FGD: Focus group discussion
MST: Marie Stopes Tanzania
NGO: Non-governmental organization
