# Supplementary material for: Getting more than “claps”: incentive preferences of voluntary community-based mobilizers in Tanzania
Source: Hum Resour Health. 2019 Dec 17;17:101. doi: 10.1186/s12960-019-0438-5 (PMC6918602; doi:10.1186/s12960-019-0438-5)
Supplement: Supplementary file 1 — Additional file 1. DCE questionnaire. [file 12960_2019_438_MOESM1_ESM.pdf]

Respondent details

Age		
Sex		
Facility		
Job	☐	CBM
	☐	CM
	☐	CAA

- You are asked 8 questions.
- For each question, choose option A or B.
- Each option contains possible conditions to support your work as a CBM.
- You always have to choose **one** of the two options. Even if you are not sure, select the option that is best according to your opinio
- Do not select both options or leave both options blank.

Question 1

Option 1.A ☐	
Remuneration	60.000 TSH/month
Training	CBMs to attend a seminar at the centre on a specific health topic every 6 months
Supervision	Monthly progress review meetings including receiving monthly progress report + centre manager or centre administrative assistant accompany all vikundi visits and give immediate feedback to CBMs
Benefits	Once every 6 months, the best performing group of CBMs in Tanzania gets a health insurance covering Marie Stopes services of 50,000 TSH for each person
Identification	CBMs will receive two blue Marie Stopes T-shirts + Marie Stopes bag once

Option 1.B ☐	
Remuneration	60.000 TSH/month
Training	CBMs to attend a seminar at the centre on a specific health topic every 2 months
Supervision	Monthly progress review meetings + MST Dar Office visits CBMs in the field every 6 months and a group picture is taken
Benefits	Once every 6 months, the best performing group of CBMs in Tanzania gets a health insurance covering Marie Stopes services of 50,000 TSH for each person
Identification	CBMs will receive two blue Marie Stopes T-shirts + Marie Stopes bag once

Question 2

Option 2.A ☐	
Remuneration	50.000 TSH/month + 10.000 TSH extra for every 5 CPAC clients referred by the CBM group
Training	CBMs to attend a seminar at the centre on a specific health topic every 6 months
Supervision	Monthly progress review meetings + MST Dar Office visits CBMs in the field every 6 months and a group picture is taken
Benefits	Once every 6 months, the best performing group of CBMs in Tanzania gets a health insurance covering Marie Stopes services of 50,000 TSH for each person
Identification	CBMs will receive two blue Marie Stopes T-shirts + Marie Stopes bag once

Option 2.B ☐	
Remuneration	50.000 TSH/month + 10.000 TSH extra for every 5 CPAC clients referred by the CBM group
Training	CBMs to attend a seminar at the centre on a specific health topic every 2 months
Supervision	Monthly progress review meetings + MST Dar Office visits CBMs in the field every 6 months and a group picture is taken
Benefits	Once every 6 months, the best performing CBM at your centre gets the opportunity to visit another centre
Identification	CBMs will receive two blue Marie Stopes T-shirts + Marie Stopes bag + ID card once

Question 3

Option 3.A ☐	
Remuneration	50.000 TSH/month + 10.000 TSH extra for every 5 CPAC clients referred by the CBM group
Training	CBMs to attend a seminar at the centre on a specific health topic every 2 months
Supervision	Monthly progress review meetings + MST Dar Office visits CBMs in the field every 6 months and a group picture is taken
Benefits	Once every 6 months, the best performing CBM at your centre gets the opportunity to visit another centre
Identification	CBMs will receive two blue Marie Stopes T-shirts + Marie Stopes bag once

Option 3.B ☐	
Remuneration	50.000 TSH/month + 10.000 TSH extra for every 5 CPAC clients referred by the CBM group
Training	CBMs to attend a seminar at the centre on a specific health topic every 6 months
Supervision	Monthly progress review meetings + MST Dar Office visits CBMs in the field every 6 months and a group picture is taken
Benefits	Once every 6 months, the best performing group of CBMs in Tanzania gets a health insurance covering Marie Stopes services of 50,000 TSH for each person
Identification	CBMs will receive two blue Marie Stopes T-shirts + Marie Stopes bag + ID card once

Question 4

Option 4.A ☐	
Remuneration	50.000 TSH/month + 10.000 TSH extra for every 5 CPAC clients referred by the CBM group
Training	CBMs to attend a seminar at the centre on a specific health topic every 2 months
Supervision	Monthly progress review meetings including receiving monthly progress report + centre manager or centre administrative assistant accompany all vikundi visits and give immediate feedback to CBMs
Benefits	Once every 6 months, the best performing group of CBMs in Tanzania gets a health insurance covering Marie Stopes services of 50,000 TSH for each person
Identification	CBMs will receive two blue Marie Stopes T-shirts + Marie Stopes bag + ID card once

Option 4.B ☐	
Remuneration	50.000 TSH/month + 10.000 TSH extra for every 5 CPAC clients referred by the CBM group
Training	CBMs to attend a seminar at the centre on a specific health topic every 2 months
Supervision	Monthly progress review meetings including receiving monthly progress report + centre manager or centre administrative assistant accompany all vikundi visits and give immediate feedback to CBMs
Benefits	Once every 6 months, the best performing group of CBMs in Tanzania gets a health insurance covering Marie Stopes services of 50,000 TSH for each person
Identification	CBMs will receive two blue Marie Stopes T-shirts + Marie Stopes bag once

Question 5

	Option 5.A ☐	Option 5.B ☐
Remuneration	60.000 TSH/month	60.000 TSH/month
Training	CBMs to attend a seminar at the centre on a specific health topic every 2 months	CBMs to attend a seminar at the centre on a specific health topic every 6 months
Supervision	Monthly progress review meetings + MST Dar Office visits CBMs in the field every 6 months and a group picture is taken	Monthly progress review meetings including receiving monthly progress report + centre manager or centre administrative assistant accompany all vikundi visits and give immediate feedback to CBMs
Benefits	Once every 6 months, the best performing group of CBMs in Tanzania gets a health insurance covering Marie Stopes services of 50,000 TSH for each person	Once every 6 months, the best performing group of CBMs in Tanzania gets a health insurance covering Marie Stopes services of 50,000 TSH for each person
Identification	CBMs will receive two blue Marie Stopes T-shirts + Marie Stopes bag + ID card once	CBMs will receive two blue Marie Stopes T-shirts + Marie Stopes bag + ID card once

Question 6

	Option 6.A ☐	Option 6.B ☐
Remuneration	50.000 TSH/month + 10.000 TSH extra for every 5 CPAC clients referred by the CBM group	60.000 TSH/month
Training	CBMs to attend a seminar at the centre on a specific health topic every 6 months	CBMs to attend a seminar at the centre on a specific health topic every 2 months
Supervision	Monthly progress review meetings including receiving monthly progress report + centre manager or centre administrative assistant accompany all vikundi visits and give immediate feedback to CBMs	Monthly progress review meetings including receiving monthly progress report + centre manager or centre administrative assistant accompany all vikundi visits and give immediate feedback to CBMs
Benefits	Once every 6 months, the best performing CBM at your centre gets the opportunity to visit another centre	Once every 6 months, the best performing CBM at your centre gets the opportunity to visit another centre
Identification	CBMs will receive two blue Marie Stopes T-shirts + Marie Stopes bag + ID card once	CBMs will receive two blue Marie Stopes T-shirts + Marie Stopes bag + ID card once

Question 7

	Option 7.A ☐	Option 7.B ☐
Remuneration	60.000 TSH/month	60.000 TSH/month
Training	CBMs to attend a seminar at the centre on a specific health topic every 6 months	CBMs to attend a seminar at the centre on a specific health topic every 6 months
Supervision	Monthly progress review meetings + MST Dar Office visits CBMs in the field every 6 months and a group picture is taken	Monthly progress review meetings + MST Dar Office visits CBMs in the field every 6 months and a group picture is taken
Benefits	Once every 6 months, the best performing CBM at your centre gets the opportunity to visit another centre	Once every 6 months, the best performing CBM at your centre gets the opportunity to visit another centre
Identification	CBMs will receive two blue Marie Stopes T-shirts + Marie Stopes bag + ID card once	CBMs will receive two blue Marie Stopes T-shirts + Marie Stopes bag once

Question 8

	Option 8.A ☐	Option 8.B ☐
Remuneration	60.000 TSH/month	50.000 TSH/month + 10.000 TSH extra for every 5 CPAC clients referred by the CBM group
Training	CBMs to attend a seminar at the centre on a specific health topic every 2 months	CBMs to attend a seminar at the centre on a specific health topic every 6 months
Supervision	Monthly progress review meetings including receiving monthly progress report + centre manager or centre administrative assistant accompany all vikundi visits and give immediate feedback to CBMs	Monthly progress review meetings including receiving monthly progress report + centre manager or centre administrative assistant accompany all vikundi visits and give immediate feedback to CBMs
Benefits	Once every 6 months, the best performing CBM at your centre gets the opportunity to visit another centre	Once every 6 months, the best performing CBM at your centre gets the opportunity to visit another centre
Identification	CBMs will receive two blue Marie Stopes T-shirts + Marie Stopes bag once	CBMs will receive two blue Marie Stopes T-shirts + Marie Stopes bag once

THANK YOU!
